# Different Patterns of Inappropriate Antimicrobial Use in Surgical and Medical Units at a Tertiary Care Hospital in Switzerland: A Prevalence Survey

**DOI:** 10.1371/journal.pone.0014011

**Published:** 2010-11-16

**Authors:** Alexia Cusini, Silvana K. Rampini, Vineeta Bansal, Bruno Ledergerber, Stefan P. Kuster, Christian Ruef, Rainer Weber

**Affiliations:** 1 Division of Infectious Diseases and Hospital Epidemiology, University Hospital Zurich, Zurich, Switzerland; 2 Division of Internal Medicine, University Hospital Zurich, Zurich, Switzerland; University of California Los Angeles, United States of America

## Abstract

**Background:**

Unnecessary or inappropriate use of antimicrobials is associated with the emergence of antimicrobial resistance, drug toxicity, increased morbidity and health care costs. Antimicrobial use has been reported to be incorrect or not indicated in 9–64% of inpatients. We studied the quality of antimicrobial therapy and prophylaxis in hospitalized patients at a tertiary care hospital to plan interventions to improve the quality of antimicrobial prescription.

**Methodology/Principal Findings:**

Experienced infectious diseases (ID) fellows performed audits of antimicrobial use at regular intervals among all patients—with or without antimicrobials—hospitalized in predefined surgical, medical, haemato-oncological, or intensive care units. Data were collected from medical and nursing patient charts with a standardized questionnaire. Appropriateness of antimicrobial use was evaluated using a modified algorithm developed by Gyssens et al.; the assessment was double-checked by a senior ID specialist.

We evaluated 1577 patients of whom 700 (44.4%) had antimicrobials, receiving a total of 1270 prescriptions. 958 (75.4%) prescriptions were for therapy and 312 (24.6%) for prophylaxis. 37.0% of therapeutic and 16.6% of prophylactic prescriptions were found to be inappropriate. Most frequent characteristics of inappropriate treatments included: No indication (17.5%); incorrect choice of antimicrobials (7.6%); incorrect application of drugs (9.3%); and divergence from institutional guidelines (8%). Characteristics of inappropriate prophylaxes were: No indication (9%); incorrect choice of antimicrobials (1%); duration too long or other inappropriate use (6.7%). Patterns of inappropriate antimicrobial varied widely in the different hospital units; empirical prescriptions were more frequently incorrect than prescriptions based on available microbiological results.

**Conclusions/Significance:**

Audits of individual patient care provide important data to identify local problems in antimicrobial prescription practice. In our study, antimicrobial prescriptions without indication, and divergence from institutional guidelines were frequent errors. Based on these results, we will tailor education, amend institutional guidelines and further develop the infectious diseases consultation service.

## Introduction

Antimicrobial therapy and prophylaxis in hospitals has been reported to be incorrect or not indicated in 9 to 64% [1]–[16] (table S1). Reasons for inappropriate prescriptions include uncertainty of differential diagnoses; complex co-morbidities; lack of training, experience, or confidence of physicians in charge; lack of knowledge of local epidemiology of antimicrobial resistance; or wrong interpretation of microbiological results, e.g. treatment of colonising bacteria. Consequences of excessive or inappropriate antimicrobial use are increased morbidity, mortality, and health care costs, because of the emergence or selection of resistant microorganisms; increased rate of *Clostridium difficile* infection, antimicrobial drug toxicity; drug-drug interactions; catheter-related infections associated with intravenous administration of antimicrobials; other nosocomial infections; and prolonged hospitalisation [17]–[23].

Antimicrobial stewardship programmes aim to reduce and optimize antimicrobial use in order to prevent the emergence of resistance or other adverse effects, improve outcome of care, and reduce health care costs without compromising quality of care [24]. Methods of such programmes include: Quantitative monitoring of antimicrobial use in hospitals or hospital units; qualitative evaluation of antimicrobial treatment and prophylaxis in individual patients; monitoring of local antimicrobial susceptibility; promotion of institutional guidelines; and education [24]–[27].

Qualitative assessment of antimicrobial use is not widely done because of lack of standardization, methodological challenges, and demanding use of human resources. However, acceptance of and motivation for education of prescribers may be encouraged by using local quality data, including examples of individual patient care. Thus, surveys of local prescribing practice among physicians working in different fields of medicine and in different hospital units may be a source for improving local institutional guidelines and tailoring interventions to foster rational and responsible antimicrobial use.

We studied the quality of antimicrobial therapy and prophylaxis among individual patients hospitalised at a tertiary care university hospital in Switzerland by repeated prevalence survey in surgical, medical and haemato-oncology wards, and in surgical and medical intensive care units (ICUs).

## Methods

### Hospital setting

The University Hospital Zurich is an 800-bed tertiary care teaching hospital. It covers all specialties except paediatrics and orthopaedics. In 2008, there were 32,724 admissions; the average length of stay was 8.2 days; and the average use of antimicrobial agents was 79.8 DDD (defined daily doses) per 100 bed-days and 554.67 DDD per 100 admissions.

Antimicrobials are prescribed by physicians in charge, based on their own decision, or based on recommendations of infectious diseases (ID) physicians, if an ID consultation was requested. The ID consultation service is available 24 hours a day, 7 days a week. Institutional guidelines on antimicrobial use were developed in 1999 in collaboration with representatives of all medical specialties and microbiologists, based on international evidence-based guidelines, local resistance data, costs, and availability of antimicrobial drugs. The guidelines were regularly updated thereafter, the current version was updated in March 2008. The guidelines recommend restrictive use of broad-spectrum antibiotics, of antibiotics associated with the emergence of resistance, of potentially toxic, or of costly antimicrobials. No mechanisms to control adherence to guidelines have been implemented so far.

### Study population

We evaluated all patients hospitalized in surgical, medical, oncology and haematology wards, as well as in the surgical and medical ICUs, excluding patients following lung transplantation. The surgical wards are divided into thoracic, cardiovascular, trauma, visceral & transplantation, and plastic & reconstructive surgery. The medical wards are divided into general internal medicine, oncology, cardiology, gastroenterology & hepatology, nephrology, haematology, pneumology, endocrinology & diabetes & clinical nutrition, angiology, and infectious diseases. The ICUs are divided in medical, neurosurgery, trauma, visceral, thoracic & transplant surgery, cardiovascular surgery, and burn ICU.

### Study design

We performed a repeated prevalence survey, evaluating all patients, with and without therapeutic or prophylactic antimicrobials, who were registered at a unit at 8 a.m. on the day of an audit. The audits were performed in the surgical wards during 10 weeks between June and August 2008, in the ICUs during 9 weeks between October and December 2008, and in the medical wards during 9 weeks between January and March 2009. In these units, one audit per week was performed and each patient was only evaluated once. In the haematology and oncology wards, the audits were performed during 10 weeks between April and June 2009; patients were evaluated repeatedly during their whole hospitalisation period at intervals of one week because antimicrobial use changed when neutropenia developed in the course of chemotherapy. Data collection and analysis as well as reporting of data to surveyed hospital units took approximately 14 months of full-time work of one ID fellow. Additionally, a senior ID specialist and a statistician supervised the project.

### Data collection

The following patients' variables were recorded: date of audit, date of birth, sex, date of hospitalisation, ward, co-morbidity (estimated using the Charlson's co-morbidity index by adding one to six points for the presence of defined diagnoses [28]), surgical procedures, ICU stay during hospitalisation, current immunosuppression, and previous consultation by an ID specialist during hospitalisation.

In patients with current antimicrobials, we recorded all prescribed antimicrobial agents (ATC group ‘J’ [29], including antibiotic, antimycobacterial, antifungal, antiviral, and antiparasitic agents), dose, route of administration, date of first prescription, and whether the drugs were given in prophylactic or therapeutic purpose.

Prescriptions were classified as ‘empirical’ when the pathogen was unknown at the time of prescription, as ‘directed’ when the pathogen was suspected based on provisional microbiological result (such as Gram stain), and as ‘targeted’ when a pathogen was identified.

Furthermore, the results of microbiological, radiological and pathological investigations available at the time of the survey were reviewed to assess the appropriateness of diagnoses of infectious diseases leading to the prescriptions of antimicrobials.

All data were collected by patient chart review; ID study physicians did not have patient contact in the context of the audits. Of note, all records of physicians and nurses, and all findings of laboratory, radiological, microbiological or other examinations were available in the electronic patient charts.

### Assessment of appropriateness of antibiotic use

The appropriateness of antimicrobial prescriptions was evaluated according to local and international evidence-based guidelines, and considering local epidemiology of antimicrobial resistance, microbiological findings, if available, and co-morbidity. The appropriateness of each prescription was assessed by two ID physicians; the first judgement was performed by an experienced ID fellow (AC, SR and VB, respectively), and was then discussed with a senior ID specialist (RW, or other ID staff physicians).

We classified the appropriateness of antimicrobial treatment (AMT) using a standardized algorithm initially reported by Gyssens et al., and modified by Willemsen and colleagues [4], [30]. This algorithm was chosen as a validated method that allows to systematically evaluate all aspects of antimicrobial prescription. In brief, AMT was judged as follows:

- Appropriate decisions; all criteria of correct antimicrobial use are fulfilled.
- Inappropriate indication; prescription of antimicrobials without the presence of an infectious disease, or prescription of antimicrobials for an infection that does not need antimicrobial treatment.
- Inappropriate choice, including inappropriate spectrum of the antimicrobial agent (too broad, too narrow, not effective), or inappropriate toxicity profile.
- Inappropriate application; including inappropriate dosage, timing, route of administration and duration of therapy.
- Divergence from guidelines.
- Missing, or insufficient data to judge the appropriateness of antimicrobial use.

### Ethics

Approval by the local ethics committee (address: Kantonale Ethikkommission Zürich, Sonneggstrasse 12, CH-8091 Zürich, Switzerland) was obtained. The ethics committed decided that patients' informed consent was not required because this study was a quality control project.

The heads of the various clinics and their staff physicians were prospectively informed about the study, and accepted the evaluation methods.

Prescribers were immediately contacted personally after review of patients' antimicrobial therapy if current prescription appeared to have imminent negative consequences for a patient in view of the evaluating ID physicians.

### Statistical analyses

All data were entered into an EpiData database, and analysed with EpiData Analysis V2 (www.epidata.dk).

## Results

### Patient characteristics

The demographic and clinical characteristics of study participants are summarised in Table 1. Between June 2008 and March 2009 a total of 1577 patients were included in the study; 543 patients were registered on the surgical wards, 553 on the medical wards, 187 on the haemato-oncology wards, and 294 on the ICUs. The mean age was 57.6 years (range: 16–98), 952 (60.4%) were male. The proportion of immunocompromised patients was highest with 73.8% in the haemato-oncology wards. Similarly, patients on the haemato-oncology wards had the highest Charlson comorbidity score with a mean of 3.68 (range 2–10). Patients on the ICUs had a mean comorbidity score of 3.27 (range 0–11).

**Table 1:** Patient characteristics.

Characteristics	Total	Surgical wards	Medical wards	ICU	Haemato-oncology wards
No. of evaluated patients (%)	1577 (100)	543 (34.4)	553 (35.1)	294 (18.6)	187 (11.9)
Female	625 (39.6)	248 (45.7)	211 (38.2)	101 (34.4)	65 (34.8)
Male	952 (60.4)	295 (54.3)	342 (61.8)	193 (65.6)	122 (65.2)
Mean age (range)	57.6 (16–98)	56.0 (16–95)	60.4 (16–98)	58.5 (15–94)	55.9 (17–90)
Mean days of hospital stay at time of evaluation (range)	7.2 (0–180)	5.0 (0–111)	6.8 (0–180)	6.0 (0–102)	7.8 (1–71)
No. of patients with ICU stay during hospitalisation (%)	508 (32.2)	136 (25.0)	68 (12.3)	294 (100)	10 (5.3)
No. of immunocompromised patients^1^ (%)	366 (23.2)	41 (7.6)	150 (27.1)	37 (12.6)	138 (73.8)
Mean Charlson comorbidity index (range)	2.7 (0–11)	1.76 (0–11)	2.99 (0–10)	3.27 (0–11)	3.68 (2–10)
Patient no. with comorbidity index = 0 (%)	354 (22.5)	248 (45.7)	66 (11.9)	40 (1.6)	0 (0)
Comorbidity index = 1–2	552 (35.0)	150 (27.6)	206 (37.3)	97 (33.0)	99 (52.9)
Comorbidity index = 3–4	288 (18.3)	61 (11.2)	138 (25.0)	68 (23.1)	21 (11.2)
Comorbidity index = ≥5	383 (24.3)	84 (15.5)	143 (25.9)	89 (30.3)	67 (35.8)
Patients on antimicrobials (%)	700 (44.4)	196 (36.1)	255 (46.1)	142 (48.3)	107 (57.2)
Patients on therapy	568 (36.0)	167(30.8)	208 (37.6)	113 (38.4)	80 (42.8)
Patients on prophylaxis	228 (14.5)	33 (6.1)	88 (15.9)	38 (12.9)	69 (36.9)
Total no. of prescriptions (%)	1270 (100)	258 (20.3)	460 (36.2)	224 (17.6)	328 (25.8)
Prescriptions for therapy	958 (75.4)	211 (81.8)	354 (77)	179 (79.9)	214 (65.2)
Prescriptions for prophylaxis	312 (24.6)	47 (18.2)	106 (23.0)	45 (20.1)	114 (34.8)
Prescriptions for surgical prophylaxis	60 (19.2)	20 (42.5)	16 (15.1)	24 (53.3)	0 (0)
Prescriptions for prophylaxis in immunocompromised patients	252 (80.8)	27 (57.5)	90 (84.9)	21 (46.7)	114 (100)

1 Immunosupression includes patients with transplantation, splenectomy, immunosuppressive therapy or steroids, agammaglobulinaemia and cellular immunodeficiency.

### Antimicrobial use and diagnoses

Of the 1577 evaluated patients, 700 (44.4%) had antimicrobials, and received a total of 1270 prescriptions. Thereof, 958 (75.4%) prescriptions were for therapy and 312 (24.6%) for prophylaxis. 252 (80.8%) prophylaxes were prescribed due to immunosuppression, and 60 (19.2%) prophylaxes were prescribed for surgical or other interventional procedures. 409 patients received one, 172 patients two and 119 patients three or more prescriptions simultaneously. The highest proportion of patients on antimicrobial treatment was in the haemato-oncology wards with 57.2%. In the ICUs the proportion of patients on antimicrobials was 48.3%, in the medical wards 46.1%, and in the surgical wards 36.1%.

Table 2 lists the most frequently prescribed antimicrobial agents for therapy and for prophylaxis in the different units. 39.0% of the antimicrobial agents were given in parenteral form.

**Table 2:** Top ten antimicrobial drugs for treatment in different hospital units: No. (%) of prescriptions.

Surgical wards		Medical wards		ICU		Haemato-oncology wards	
Total no. (%)	211 (100)	Total no. (%)	354 (100)	Total no. (%)	179 (100)	Total no. (%)	214 (100)
Amoxicillin-clavulanate	85 (40.3)	Piperacillin-tazobactam	58 (16.4)	Piperacillin-tazobactam	32 (17.9)	Piperacillin-tazobactam	25 (11.7)
Ciprofloxacin	32 (15.2)	Amoxicillin-clavulanate	38 (10.7)	Amoxicillin-clavulanate	31 (17.3)	Aciclovir	23 (10.7)
Piperacillin-tazobactam	19 (9.0)	Ceftriaxone	24 (6.8)	Meropenem	23 (12.8)	Meropenem	19 (8.9)
Metronidazole	14 (6.6)	Ciprofloxacin	21 (5.9)	Vancomycin	23 (12.8)	Amoxicillin-clavulanate	15 (7.0)
Rifampicin	6 (2.8)	Metronidazole	18 (5.1)	Ciprofloxacin	10 (5.6)	Ciprofloxacin	13 (6.1)
Trimethoprim-sulfamethoxazole	5 (2.4)	Valaciclovir	15 (4.2)	Ceftriaxone	8 (4.5)	Voriconazole	13 (6.1)
Ceftriaxone	4 (1.9)	Levofloxacin	10 (2.8)	Fluconazole	8 (4.5)	Caspofungin	12 (5.6)
Clindamycin	4 (1.9)	Fluconazole	9 (2.5)	Clindamycin	8 (4.5)	Metronidazole	10 (4.7)
Cefuroxime	4 (1.9)	Clarythromycin	9 (2.5)	Moxifloxacin	8 (4.5)	Cefepime	9 (4.2)
Fluconazole	3 (1.4)	Meropenem	6 (1.7)	Metronidazole	6 (3.4)	Valaciclovir	9 (4.2)
Other	35 (16.6)	Other	146 (41.2)	Other	22 (12.3)	Other	66 (30.8)

In table 3, diagnoses and main indications for therapeutic use are summarized. The indications for antimicrobial therapy varied widely between the different units because of differences in underlying diseases of patients hospitalised in these units. Overall, the most frequent diagnoses were respiratory tract infections (n = 138, 21.3%), peritonitis (n = 43, 6.6%), sepsis (n = 42, 6.5%), and skin/soft tissue infections (n = 41, 6.3%). In 72 (11.1%) patients receiving antimicrobials, no infection was present.

**Table 3:** Main indications for antimicrobial therapy.

	Total	Surgical wards	Medical wards	ICU	Oncology, haematology wards
Total no. of diagnoses (%)^1^	648 (100)	167(100)	208 (100)	113 (100)	160 (100)
Respiratory tract infection	138 (21.3)	12 (7.2)	64 (30.7)	31 (27.4)	31 (19.4)
Peritonitis	43 (6.6)	21(12.6)	7 (3.4)	12 (10.6)	3 (1.9)
Sepsis, bacteraemia	42 (6.5)	6 (3.69	9 (4.3)	20 (17.7)	7 (4.4)
Skin, soft tissue infection	41 (6.3)	10 (6.0)	23 (11.1)	2 (1.8)	6 (3.9)
Fever in neutropenia	35 (5.4)	0 (0)	7 (3.4)	0 (0)	28 (18.2)
Infection of unclear origin	34 (5.2)	7 (4.2)	9 (4.3)	6 (5.3)	12 (7.5)
Gastrointestinal tract infection	32 (4.9)	3 (1.5)	18 (8.7)	2 (1.8)	9 (5.8)
Postoperative wound infection	31 (4.8)	25 (15.0)	0 (0)	6 (5.3)	0 (0)
Traumatic wound/open fracture	29 (4.5)	24 (14.4)	0 (0)	5 (4.4)	0 (0)
Pulmonary aspergillosis	25 (3.9)	0 (0)	5 (2.4)	0 (0)	20 (13.0)
Cardiovascular infection	20 (3.1)	0 (0)	13 (6.2)	7 (6.2)	0 (0)
Urinary tract infection	19 (2.9)	0 (0)	15 (7.2)	1 (0.9)	3 (1.9)
Herpes simplex virus	19 (2.9)	0 (0)	0	0 (0)	19 (12.3)
Hepatitis	10 (1.5)	0 (0)	8 (3.8)	1 (0.9)	1 (0.6)
Infections with Candida	7(1.1)	0 (0)	0 (0)	0 (0)	7 (4.4)
Other	51 (7.9)	16 (9.6)	22 (10.6)	5 (4.4)	8 (5.0)
No infection	72 (11.1)	43 (25.7)	8 (3.8)	15 (13.3)	6 (3.8)

1 In the surgical and medical wards and ICUs we documented the leading infection whereas in the haemato-oncology wards all infections occurring during the hospitalisation were documented.

### Appropriateness of antimicrobial therapy and prophylaxis

Table 4 gives an overview of the evaluation of the appropriateness of antimicrobial use. A total of 406 (32%) prescriptions were judged as inappropriate. Therapies were more frequently inappropriate than prophylaxes (37.0% versus 16.6%).

**Table 4:** Evaluation of the appropriateness of antimicrobial treatment and prophylaxis.

			Total	Surgical wards	Medical wards	ICU	Haemato-oncology wards
**Therapy**	No. of patients with antimicrobial therapy		568	167	208	113	80
	No. of prescriptions (%)		958 (100)	211 (100)	354 (100)	179 (100)	214 (100)
	Total no. of inappropriate prescriptions		354 (37.0)	104 (49.3)	115 (32.9)	58 (32.4)	77 (36.0)
	Inappropriate indication *	Total	168 (17.5)	64 (30.3)	41 (11.6)	33 (18.4)	30 (14.0)
		No infection	101 (10.6)	57(27.0)	16 (4.5)	21 (11.7)	7 (3.2)
		Infection, no antimicrobials needed	67 (7.0)	7 (3.3)	25 (7.1)	12 (6.7)	23 (10.7)
	Inappropriate choice *	Total	73 (7.6)	23 (10.9)	30 (8.4)	14 (7.8)	6 (2.8)
		Spectrum too broad	31 (3.2)	5 (2.3)	19 (5.3)	7 (3.9)	0 (0)
		Spectrum too narrow	16 (1.7)	7 (3.3)	2 (0.6)	2 (1.1)	5 (2.3)
		Spectrum ineffective	24 (2.5)	11 (5.2)	9 (2.5)	3 (1.6)	1 (0.5)
		Inappropriate toxicity profile	2 (0.2)	0 (0)	0 (0)	2 (1.1)	0 (0)
	Inappropriate application *	Total	89 (9.3)	20 (9.5)	35 (9.9)	12 (6.7)	22 (10.3)
		Dosage	31 (3.2)	2 (9.5)	12 (3.4)	7 (3.9)	10 (4.7)
		Timing	20 (2.1)	1 (0.5)	14 (4.0)	2 (1.1)	3 (1.4)
		Route of administration	9 (0.9)	3 (1.4)	3 (0.8)	1 (0.6)	2 (1.8)
		Duration too long	29 (3.0)	14 (6.6)	6 (1.6)	2 (1.1)	7 (3.3)
	Divergence from internal guidelines		77 (8.0)	2 (0.9)	39 (11.0)	12 (6.7)	24 (11.2)
	Data insufficient for evaluation of appropriateness		23 (2.4)	0 (0)	9 (2.5)	6 (3.4)	8 (3.7)
**Prophylaxis**	No. of patients with antimicrobial prophylaxis		228	33	88	38	69
	No. of prescriptions (%)		312 (100)	47 (100)	106 (100)	45 (100)	114 (100)
	Total no. of inappropriate prescriptions		52 (16.6)	6 (12.8)	17 (16.0)	16 (35.6)	13 (11.4)
	Inappropriate indication *		28 (9.0)	1 (2.1)	10 (9.4)	8 (17.8)	9 (7.9)
	Inappropriate spectrum *		3 (1.0)	0 (0)	1 (0.9)	2 (4.4)	0 (0)
	Inappropriate application *	Total	21 (6.7)	5 (10.6)	6 (5.6)	6 (13.3)	4 (3.5)
		Duration too long	16 (5.1)	5 (10.6)	6 (5.6)	5 (11.1)	0 (0)
		Dosage/timing	4 (1.3)	0 (0)	0 (0)	1 (2.2)	4 (3.5)

*One prescription can include more than one inappropriate decision.

In 171 (27.5%) patients on antimicrobials, receiving a total of 279 therapeutic or prophylactic prescriptions, an ID consultation was performed during hospitalisation, but this consultation was not always current on the day of audit. Among patients with ID consultations, the proportion of inappropriate prescriptions was 16.5%, indicating a significantly lower rate than among patients without ID consultation.

We found 354 (37%) prescriptions of antimicrobial therapy to be inappropriate accounting for a total of 407 errors (more than one criterion defining inappropriateness was possible in one prescription). Categories of inappropriateness included: 168 (17.5%) no indication, 73 (7.6%) incorrect choice, 89 (9.3%) incorrect application, and 77 (8%) divergence from local guidelines.

In the various hospital units there were remarkable differences in the patterns of inappropriate prescribing. In surgical wards the main problem was lack of indication in 30.3% of prescriptions, particularly including pre-emptive antibiotic therapy after surgical interventions. In the medical wards, no indication was found in 11.6% of prescriptions, divergence from guidelines in 11%, and inappropriate application in 9.9%. Similar patterns of inappropriate use were detected in the haemato-oncology ward: no indication in 14%, and divergence from guidelines in 11.2%. In the ICUs, no indication was present in 18.4%, incorrect choice was observed in 7.8%, and incorrect application in 6.7% of prescriptions.

Table 5 summarizes the appropriateness with regard to empirical versus directed and versus targeted antimicrobial prescriptions. Across all wards, empirical prescriptions were judged more often as inappropriate (42.6%) than directed (22.7%) or targeted prescriptions (18.5%) (p = 0.001).

**Table 5:** Appropriateness of empiric, directed, and targeted prescriptions.

Therapy	Total	Surgical wards	Medical wards	ICU	Hemato-oncology wards
Total no. of prescriptions	958	211	354	179	214
Empiric prescriptions*	688 (100)	152 (100)	238 (100)	117 (100)	181 (100)
Inappropriate	292 (42.6)	92 (60.5)	92 (38.7)	48 (41.0)	60 (33.2)
Directed prescriptions*	97 (100)	12 (100)	75 (100)	10 (100)	n.a.**
Inappropriate	22 (22.7)	2 (16.7)	16 (21.3)	4 (40.0)	n.a.**
Targeted prescriptions*	173 (100)	47 (100)	41 (100)	52 (100)	33 (100)
Inappropriate	32 (18.5)	10(21.3)	7(17.1)	9 (17.3)	6 (18.2)

*Prescriptions were classified as ‘empirical’ when the pathogen was unknown at the time of prescription, as ‘directed’ when the pathogen was suspected based on provisional microbiological result (such as Gram stain), and as ‘targeted’ when a pathogen was identified.

**n.a. = not applicable. In the haemato-oncology wards we did not differentiate between empirical and directed therapy because in neutropenic patients empirical therapy is rarely adapted to tentative microbiological results.

55.4% of antimicrobials were prescribed within the first three days of hospitalization, presumably for community-acquired infections; and 44.6% of antimicrobials were started after the first three days of hospitalization, presumably to treat nosocomial infections. Overall the rates of inappropriateness between these two types of prescriptions were not different (37.2% versus 35.0% inappropriate, p = 0.78). However, in the haemato-oncology wards, the proportion of inappropriateness was lower for the prescriptions within 3 versus after 3 days of hospitalization (17.7% versus 35%, p = 0.43).

Incorrect use of prophylaxis was found for 52 (16.6%) prescriptions: 28 (9%) no indication, 3 (1%) antibiotic chosen did not cover the antimicrobial spectrum to be expected, and 21 (6.7%) inappropriate application.

## Discussion

We found that 44.4% of patients—hospitalised in medical, haemato-oncological, and surgical wards, and ICUs at a tertiary care hospital in Switzerland—received antimicrobials at the time of audits. Among these, we reviewed a total of 1270 antimicrobial prescriptions—958 (75.4%) for treatment and 312 (24.6%) for prophylaxis—concluding that 37.0% of the therapeutic and 16.6% of the prophylactic prescriptions were inappropriate. There were remarkable differences in the patterns of inappropriate prescribing in the different hospital units. The most frequent errors in surgical wards, medical wards, and ICUs were prescriptions without an indication, whereas divergence from local guidelines was the most important concern in haemato-oncology units. Empirical prescriptions were more often inappropriate then directed or targeted prescriptions.

The use of antimicrobial agents has widely been assessed in hospitals or hospital units by measuring quantitative pharmacy data to calculate the number of defined daily doses (DDD) per numbers of occupied bed-days [29], or per hospital admissions of patients. Results have been used for benchmark purposes, but this methodology has drawbacks [26]: First, DDD and prescribed daily doses may differ according to the underlying disease. Second, days of therapy are underestimated in case of dose reduction, e.g., because of reduced renal function. Third, the quantitative measurement does not indicate whether therapy was appropriate. Finally, patients' morbidities may be very different between hospitals or in different hospital units. Thus, in addition to quantitative data, assessment of antimicrobial therapy and prophylaxis in individual patients is needed for quality control. However, such evaluation is difficult to standardise, particularly in patients with co-morbidities; requires substantial human resources, time, and expertise; and may cause conflicts between evaluating and evaluated physicians.

Although methods for qualitative assessments of antimicrobial use have been described [30]–[33], they are not widely used. Therefore, benchmarking and comparison of results, obtained in different countries, hospitals or hospital units, appear difficult. We used an algorithm developed and used by Gyssens et al. [30]–[31], and modified by Willemsen et al. [4], which systematically documents the indication for and use of antimicrobials among patients with and without such agents. Furthermore, we determined—according to the antibiotic policy of our hospital—the adherence to our internal guidelines that we had developed in collaboration with staff of all hospital units and microbiologists.

We assessed the quality of antimicrobial prescribing in individual patients during their hospitalisation and while on antimicrobial therapy or prophylaxis. Limitations of this approach are that assessment did not take place at time of prescription, and it was based on chart review and not on clinical examination. However, the electronic chart provided comprehensive information on the course of the hospitalisation, all notes of physicians and nurses, and on-line reports on microbiological and radiological findings. We immediately contacted prescribers and asked for further information at time of evaluation if assessment of antimicrobial prescription was not possible based on patients' chart, or if current prescription of antimicrobials appeared to have imminent negative consequences for a patient in view of evaluating ID physicians. Strengths of our study are that we collected data on more than 1200 antimicrobial prescriptions that are representative for medical and surgical specialties at our hospital, including wards and ICUs with the highest use. Furthermore, we considered all classes of antimicrobials (i.e., antibiotics, antivirals, antifungals, and antiparasitics); we evaluated therapy and prophylaxis; we evaluated all aspects of prescribing (indication, choice of drug, including spectrum, dosing, duration, and route of administration); and we carefully discussed our decisions between two ID physicians.

A comparison of published results on the appropriateness of antimicrobial use in hospitalised patients in various countries and in different hospital units during the last 10 years is summarized in table S1. Inappropriate antimicrobial use, although difficult to compare because of different methods of assessment and different ways of reporting, ranged between 9% and 64%; most studies showed rates between 30 and 40%. In all studies, data were collected with structured questionnaires, and appropriateness was mostly evaluated by ID specialists. Only five reports refer to published methods for evaluation of antimicrobial use: One report [4] refers to the algorithm developed by Gyssens et al. [30]–as described in our methods section; four studies [1], [5], [9], [16] used criteria of Kunin et al. [32] or Jones et al. [33]–categorising effectiveness, toxicity, costs, length of treatment, and appropriateness of indication of antimicrobial use. Reported results do not consequently distinguish therapy and prophylaxis: Six studies evaluated therapies, nine evaluated therapies and prophylaxes, and one report did not provide this information. Several studies limited the number of analysed antimicrobial agents, or assessed antibiotics only. The prevalence of patients on antimicrobials, if reported, ranged between 15%—in a medical department [7]—to 60% in medical and surgical ICUs [8]. Quality of antimicrobial use is reported as proportion of patients with incorrect treatment in six studies [4]–[7], [10], [12]; as proportion of inappropriate prescriptions in eight studies [1], [3], [8]–[9], [13]–[16]; as proportion of inappropriately treated infections in one study [2]; and as unnecessary days of treatment in one study [11]. We preferred to report our results as proportion of inappropriate prescriptions (and not as proportion of patients) because 41.6% of our patients had more than one concurrent prescription at the time of evaluation. In addition, nine studies provide information on categories of errors, mainly including antimicrobials without indication; inappropriate choice, and incorrect use [3]–[5], [8]–[10], [14]–[15].

Our results have clinical implications at our hospital: We identified main patterns of inappropriate antimicrobial prescribing which we will use to tailor education, to amend institutional guidelines, and to further develop the ID consultation service. Major causes of antimicrobial misuse were prescriptions without indication, unnecessary pre-emptive antibiotics after surgery; extended duration of perioperative antibiotic prophylaxes; overuse of broad-spectrum antibiotics; dual antibiotic therapy with overlapping antimicrobial spectrum; missing adaptation of the dosage according to renal function; and inappropriate prescriptions following insufficient microbiologic, radiologic or other essential investigations. Furthermore, we found a high rate of non-adherence to the institutional guidelines, indicating that it is not sufficient to provide guidelines but rather necessary to promote their application. However, guidelines at our institution were developed involving all stakeholders because prescribers should accept guidelines as professional support for decision-making rather than perceive them as an externally or administratively imposed tool for cost containment. Despite continuous availability of an ID consultation service, in only 27.5% of patients on antimicrobials who were evaluated in the audits, an ID consultation had previously been requested.

In conclusion, audits of antimicrobial use – in addition to feedback from infectious diseases specialists to individual prescribers as part of the ID consultation service – are feasible, and are needed to provide detailed and representative data about specific patterns of inappropriate prescriptions at an institution. Audits of individual patient care require significant human resources that may be the limiting factor for achieving a representative sample size. Assuming binomial distributions, 30 or 44 prescriptions have to be evaluated to have a 95% or 99% chance to detect at least one error occurring with a 10% frequency. For errors of 1% occurrence, approximately 300 or 460 prescriptions, respectively, are required. In our experience, the Gyssens' algorithm is a reliable tool to measure the appropriateness of antibiotic use and we recommend using this method for further studies for evaluating antimicrobial use in individual patients. We found few results in the literature for comparison and benchmark purposes because there is no widely used structured and validated standard method for evaluating antimicrobial use in individual patients. Antimicrobials are prescribed by physicians with or without training in infectious diseases, and therefore, characterization of patterns of inappropriate antimicrobial use is important for planning of tailored education. It is, in our opinion, not required that all antimicrobials are prescribed by ID physicians. However, all prescribers must have appropriate training, particularly including education on adverse effects of antimicrobials in individual patients and on negative long-term public health consequences of antimicrobial resistance.

## Supporting Information

Table S1 Reported studies on evaluation of appropriateness of antimicrobial use between 2000 and 2009.
(0.16 MB DOC)
