# Supplementary material for: Different Patterns of Inappropriate Antimicrobial Use in Surgical and Medical Units at a Tertiary Care Hospital in Switzerland: A Prevalence Survey
Source: PLoS One. 2010 Nov 16;5(11):e14011. doi: 10.1371/journal.pone.0014011 (PMC2982822; doi:10.1371/journal.pone.0014011)
Supplement: Table S1 — Reported studies on evaluation of appropriateness of antimicrobial use between 2000 and 2009. (0.16 MB DOC) [file pone.0014011.s001.doc]

| **Country, year [reference]** | **Type of hospital; medical specialties** | **Study design** | **Methods of evaluation of antimicrobial use** | **Antimicrobials studied; indication for use** | **No. (%) of patients on antimicrobials / no. evaluated** | **No. of evaluated prescriptions** | **Proportion of inappropriateness of antimicrobial use** |
| --- | --- | --- | --- | --- | --- | --- | --- |
| Switzerland, 2010, present study | Tertiary care hospital; medical and surgical wards, ICU | Audit | ID specialist used criteria by Gyssens et al. [30], modified by [4] | All; therapy and prophylaxis | 700 (44.4) / 1577 | Total 1270 (therapy 958, prophylaxis 312) | Therapy 37%, prophylaxis 16.6% |
| Turkey, 2009 [1] | University hospital; medical and surgical wards | Cross sectional study | Kunin [32] and Jones [33] criteria | Therapy and prophylaxis | 495 (11) /4380 | 776 | 9% |
| Italy, 2008 [2] | Community hospital; general ICU | Prospective cohort study | Independent physician | Therapy | 177 (19.7) / 898 | Not reported (no. of infections reported, n=205) | 31.2% |
| France, 2007 [3] | University hospital; medical, obstetric and gynaecological wards, ICU | Prospective descriptive clinical audit | ID specialist at bedside within 12 h | Therapy | not reported | 122 | 64% |
| Netherlands, 2007 [4] | Teaching hospital; all medical specialties | 6 repeated prevalence surveys within 3 years | Criteria by Gyssens et al. [30] | Antibacterials only; therapy and prophylaxis | 938 (22.8) / 4105 | Not reported | 37.4% |
| Croatia, 2007 [5] | University hospital; department of medicine | Prospective study | Criteria by Kunin [32] | Therapy | 438 / not reported | Not reported | 64% |
| United Kingdom, 2007 [6] | 10 acute care hospitals; medical and surgical wards | Point prevalence survey | ID specialist and computerised algorithm | Intravenous-therapy | 1079 (28.3) / 3826 | 575 prescriptions of intravenous antimcirobials | 20% |
| Croatia, 2005 [7] | University hospital; department of medicine | Prospective longitudinal survey | Scoring system to asses necessity | Therapy | 493 (15) / 3356 | 827 | 29% |
| Turkey, 2005 [8] | Tertiary care hospital; surgical and medical ICU | Prospective study | Two ID specialists | Prophylaxis and treatment | 223 (60) / 368 | 440 | 47.3% |
| Turkey, 2005 [9] | Tertiary care hospital;  medical and surgical wards | Cross sectional study | Kunin [32] and Jones [33] criteria | Prophylaxis and treatment | 378 (53) / 717 | 498 | 44 % |
| Switzerland, 2004 [10] | 8 acute-care regional reference hospitals; medical and surgical wards | One day-prevalence study | ID specialist | Antibacterial agents | 173 (25) / 695 | 197 | 47% |
| USA, 2003 [11] | Tertiary care hospital; medical, surgical rehabilitation ward, subacute nursing and obstetric-gynaecologic ward | Prospective observational study | Two ID specialists | All; therapy and prophylaxis | 129 / not reported | 153 | 30% |
| UK, 2003 [12] | Teaching hospital; acute medicines assessment unit | Prospective study | ID specialist | Antibiotics; therapy | 221 (17) / 1303 | Not reported | 48% |
| Turkey, 2003 [13] | Tertiary care hospital; medical, surgical wards, ICUs | Cross sectional study | Two ID specialists | Antibiotics; therapy and prophylaxis | 281 (39) / 713 | 377 | 35.8% |
| France, 2003 [14] | Not reported; department of internal medicine-infectious diseases | Audit study | Two senior ID specialist | Therapy and prophylaxis | 105 (53) / 198 | 105 | 35% |
| Israel, 2001 [15] | University hospital; medical geriatric, emergency paediatric, gynaecology, surgery, urology, orthopaedics, ICU | Longitudinal surveillance | Compliance with internal guidelines | Therapy and prophylaxis, (excluding preoperative prophylaxis) | 2306 /not reported | 6376 | 20% |
| Turkey, 2000 [16] | University hospital; medical and surgical wards | Cross sectional study | Kunin [32] and Jones [33] criteria | Therapy and prophylaxis (excluding preoperative prophylaxis) | 156 (17) / 937 | 234 | 54.3% |
